# Near-infrared (NIR) up-conversion optogenetics

**DOI:** 10.1038/srep16533

**Published:** 2015-11-10

**Authors:** Shoko Hososhima, Hideya Yuasa, Toru Ishizuka, Mohammad Razuanul Hoque, Takayuki Yamashita, Akihiro Yamanaka, Eriko Sugano, Hiroshi Tomita, Hiromu Yawo

**Affiliations:** 1 Department of Developmental Biology and Neuroscience, Tohoku University Graduate School of Life Sciences, Sendai 980-8577, Japan; 2 Graduate School of Bioscience and Biotechnology, Tokyo Institute of Technology, Yokohama 226-8501, Japan; 3 Department of Neuroscience II, Research Institute of Environmental Medicine, Nagoya University, Furo-cho, Chikusa-ku, Nagoya, 464-8601, Japan; 4 Laboratory of Visual Neuroscience, Department of Chemistry and Bioengineering, Iwate University Graduate School of Engineering, 4-3-5 Ueda, Morioka, Iwate 020-8551, Japan; 5 Clinical Research, Innovation and Education Center, Tohoku University Hospital, 1-1 Seiryo, Aoba, Sendai, Miyagi 980-8574, Japan; 6 Center for Neuroscience, Tohoku University Graduate School of Medicine, Sendai 980-8575, Japan

## Abstract

Non-invasive remote control technologies designed to manipulate neural functions have been long-awaited for the comprehensive and quantitative understanding of neuronal network in the brain as well as for the therapy of neurological disorders. Recently, it has become possible for the neuronal activity to be optically manipulated using biological photo-reactive molecules such as channelrhodopsin (ChR)-2. However, ChR2 and its relatives are mostly reactive to visible light, which does not effectively penetrate through biological tissues. In contrast, near-infrared (NIR) light (650–1450 nm) penetrates deep into the tissues because biological systems are almost transparent to light within this so-called ‘imaging window’. Here we used lanthanide nanoparticles (LNPs), composed of rare-earth elements, as luminous bodies to activate ChRs since they absorb low-energy NIR light to emit high-energy visible light (up-conversion). Here, we created a new type of optogenetic system which consists of the donor LNPs and the acceptor ChRs. The NIR laser irradiation emitted visible light from LNPs, then induced the photo-reactive responses in the near-by cells that expressed ChRs. However, there remains room for large improvements in the energy efficiency of the LNP-ChR system.

Non-invasive remote control technologies designed to manipulate neural functions in the brain have been long-awaited for advancing a comprehensive and quantitative understanding of the neuronal network in the brain as well as for the therapy of neurological disorders such as Parkinson’s disease. Using optogenetics, target neurons have been genetically engineered to express light-sensitive proteins such as channelrhodopsin (ChR)-2^1^, one of the algal photoreceptor proteins, and controlled to become active under irradiation by a light-emitting diode (LED) or a laser^2^,^3^. Alternatively, they have been expressed with the Cl^−^/H^+^ transporter rhodopsins, such as halorhodopsin (Halo/NpHR) and archaerhodopsin (Arch/aR-3, ArchT), to be silenced by light^4^,^5^,^6^,^7^. Nowadays, optogenetics has become widely used because of its high spatiotemporal precision resulting from the development of various molecular tools and gene-delivery methods^8^,^9^. However, the neuronal manipulations *in vivo* brain have been performed experimentally with light sources such as an LED and a laser that are connected to the targeting regions through an optic cable because of the tissue absorption of visible light^10^,^11^. The optic cables and the large LED or laser are inconvenient for experiments with free-moving animals or to deliver the light as a medical treatment. Therefore, one challenge is to invent smart, wireless implants with a remote controlling system^12^,^13^,^14^,^15^.

Near-infrared (NIR) light (650–1450 nm) penetrates deep into the tissues because biological systems are almost transparent to light within this so-called ‘imaging window’^16^. Recently, ChRs responsive to red light have been obtained through gene mining or molecular modifications^17^,^18^. For example, neurons deep in the mouse brain were activated by red light even through skin and skull when they expressed ReaChR, a variant C1V1 which is a chimera of ChR1 and one of *Volvox carteri*-derived ChRs (VChR1). However, no biological molecules known at present are optimized to absorb NIR light. Here, we have the idea of using lanthanide nanoparticles (LNPs), composed of rare-earth elements, as luminous bodies to activate ChRs since they absorb low-energy photons of NIR light to emit high-energy photons of visible light (up-conversion)^19^. A preliminary report of this research has been published elsewhere^20^.

## Results

**Figure 1:**
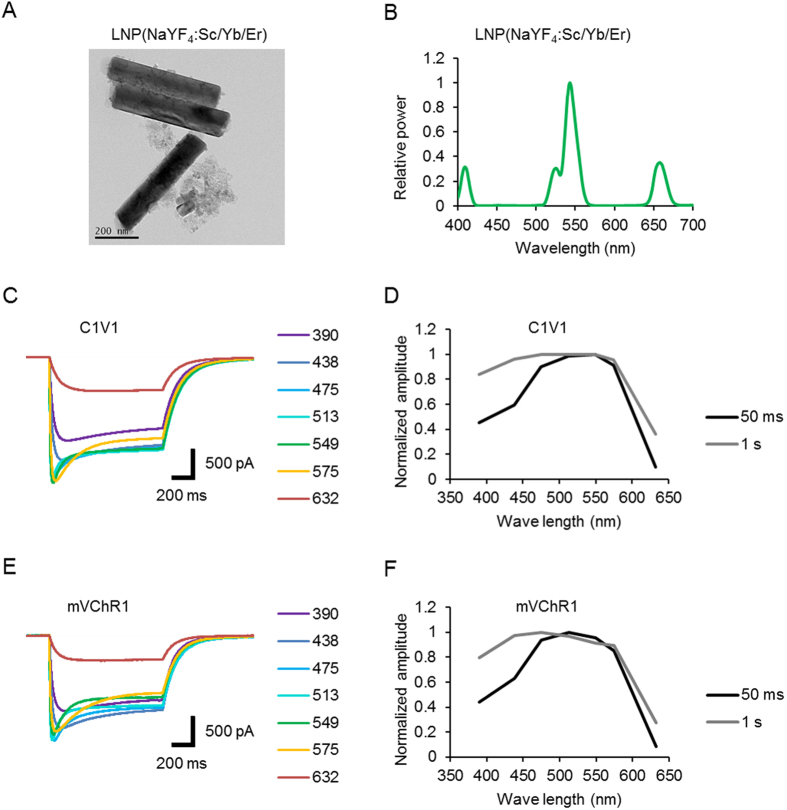
Donor-acceptor matching between LNP(NaYF4:Sc/Yb/Er) and ChRs. (**A**) TEM images of LNP(NaYF_4_:Sc/Yb/Er). Scale, 200 nm. The rod-shaped materials were made of the hexagonal-phase crystals grown from the cubic phase crystals seen as smaller particles in the TEM image. These two species were inseparable. The relatively strong emission of LNP was mainly due to the hexagonal-phase crystals. (**B**) The relative emission spectrum. (**C**) Sample photocurrents of C1V1. Each photocurrent was measured from a ND7/23 cell expressing C1V1 in response to 1-s irradiation of light at 390, 438, 475, 513, 549, 575 or 632 nm. Each power of light was adjusted to 0.5 mW/mm^2^ on the specimen. (**D**) Spectral sensitivity of C1V1 photocurrent; charge during initial 50 ms (black) and 1 s (gray). (**E**) Sample photocurrents of mVChR1. Each photocurrent was measured from a ND7/23 cell expressing mVChR1 in response to 1-s irradiation of light at 390, 438, 475, 513, 549, 575 or 632 nm. Each power of light was adjusted to 0.5 mW/mm^2^ on the specimen. (**F**) Spectral sensitivity of mVChR1 photocurrent; charge during initial 50 ms (black) and 1 s (gray).

One of the LNPs, LNP(NaYF_4_:Sc/Yb/Er), absorbs NIR of about 975 nm and emits visible light with several peaks, with the maximal peak at 543 nm (Fig. 1A,B). Therefore, the green light-absorbing ChRs would be optimal as an acceptor when LNP(NaYF_4_:Sc/Yb/Er) is used as the donor. The chimeric variants of VChR1, such as C1V1^21^ and mVChR1^22^, were the acceptor candidates because of their relatively high sensitivity to green light (Fig. 1C–F). The total charge between 0 and 50 ms peaked at 549 nm for C1V1 and at 513 nm for mVChR1. However, those between 0 and 1 s were less dependent on the spectrum due to the relatively large off time constants of C1V1 (150 ± 9.9 ms, n = 8) and mVChR1 (100 ± 7.3 ms, n = 8).

**Figure 2:**
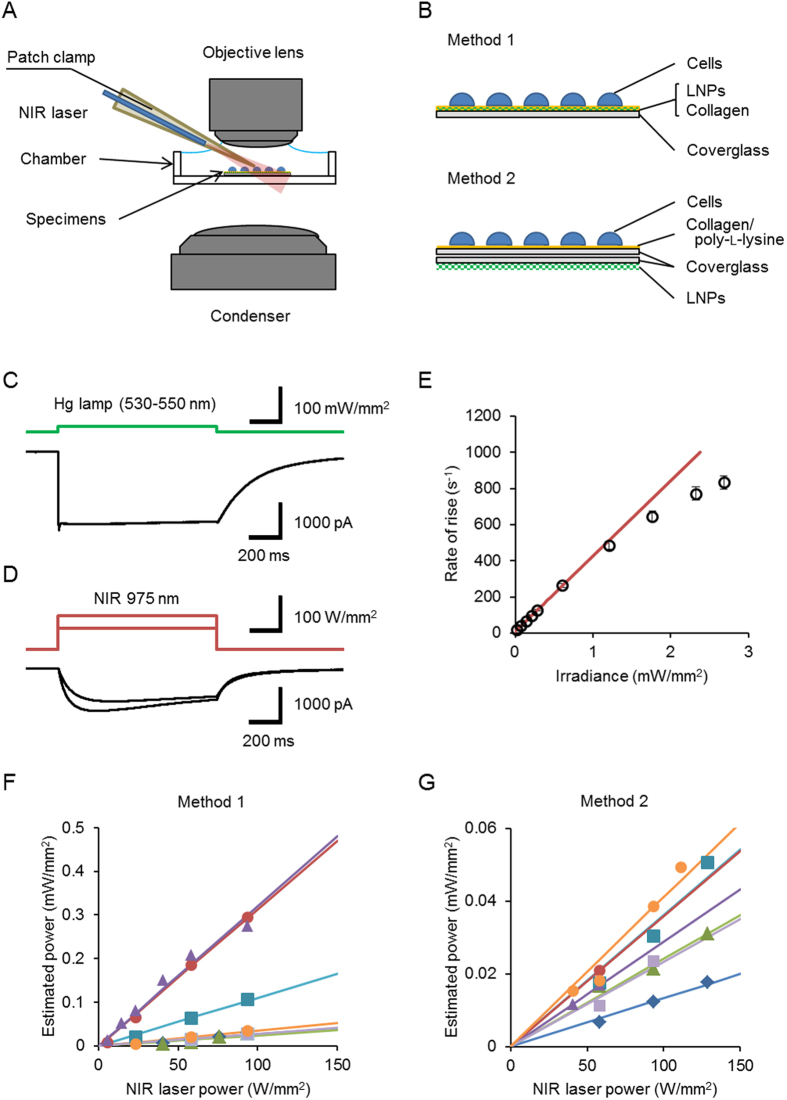
Near-infrared (NIR) up-conversion activation of C1V1. (**A**) Experimental setup. The NIR laser light (975 nm) was irradiated from a fiber (diameter, 50 μm) in the patch electrode at a distance of 5.0 mm from the tip. (**B**) Two methods to apply LNPs to the specimens. Method 1: cells were cultured on a mixture of collagen and LNP on a coverslip. Method 2: the LNP-coated coverslip was put underneath the specimens upside down in the recording chamber. (**C**) A sample photocurrent of C1V1 evoked by filtered Hg lamp (530–550 nm, 14 mW/mm^2^) at a holding potential of −60 mV (ND7/23 cells). (**D**) In the same cell, photocurrents were also evoked by the NIR laser light (975 nm, 58 and 94 W/mm^2^). (**E**) Rate of rise of C1V1 photocurrent as a function of irradiance at 549 nm. Each symbol is a mean ± SEM (n = 5). The red line is drawn by the least squares fitting to the experimental responses to light less than 1 mW/mm^2^: *y* = 420*x* + 8.8 or *x* = 0.0024*y* − 0.021. (**F**) The relationship between the estimated power of green light emission and the NIR laser power in the case of Method 1. Each symbol represents individual ND7/23 cell. The slope of linear approximation gives the energy efficiency. (**G**) Similar to F, but in the case of Method 2. Note that the scale of ordinate is different.

The LNP particles were set close to the recording cell by either Method 1 or 2 (Fig. 2A,B, see Methods section). When a C1V1-expressing ND7/23 cell was irradiated with filtered green light of a Hg lamp (530–550 nm; 14 mW/mm^2^), an almost maximal inward photocurrent was generated with a peak and plateau at a holding potential of −60 mV (Fig. 2C). Inward currents were also evoked in the same cell by the NIR laser light (975 nm) in a manner dependent on the power (Fig. 2D). In summary, in the experiments using Method 1, the NIR laser light at 58 W/mm^2^ evoked 70 ± 8.0 % (n = 7) of the peak and 59 ± 6.8 % (n = 7) of the steady state of the maximal photocurrent evoked by the Hg lamp. On the other hand, in the experiments using Method 2, the same power of NIR irradiation evoked peak and steady-state photocurrents of 38 ± 2.6% (n = 7) and 38 ± 4.2% (n = 7) of the maximum, respectively. The difference between Method 1 and 2 was significant for both peak and steady-state photocurrents (P < 0.05, Mann-Whitney *U*-test). The rate of rise of the C1V1 photocurrent was a function of the irradiance at 549 nm and was approximated to the first order relationship for the relatively small irradiance (<1 mW/mm^2^) (Fig. 2E). As the rates of rise of the above samples ranged between 12 and 95 s^−1^, the power of the emitted light was estimated from the rate of rise using this first order relationship. The estimated power of green emission was variable from one cell to another but almost proportional to the NIR laser power in the same cell using either Method 1 (Fig. 2F) or Method 2 (Fig. 2G). Each slope gave the energy efficiency that is the quotient of the visible light power received by a cell divided by the NIR laser power. The energy efficiency was on average 0.12 × 10^−3^ ± 0.052 × 10^−3^% (range, 0.023 × 10^−3^–0.32 × 10^−3^%, n = 7) by Method 1 and 0.029 × 10^−3^ ± 0.0036 × 10^−3^% (range, 0.013 × 10^−3^–0.041 × 10^−3^%, n = 7) by Method 2 with insignificant difference (Supplementary Fig. S1 online). The variability was larger in Method 1 (coefficient of variation, 1.1) than in Method 2 (coefficient of variation, 0.32).

**Figure 3:**
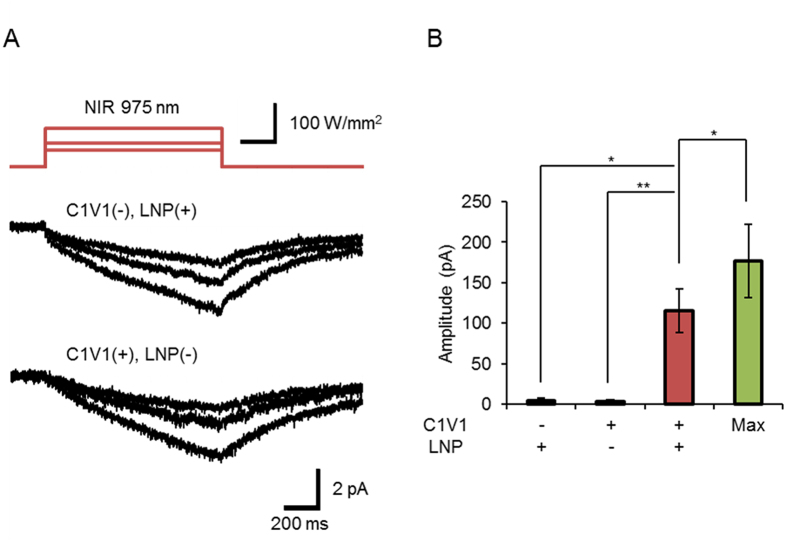
Direct effects of NIR light. (**A**) Top, the NIR laser at 41, 58 and 94 W/mm^2^. Middle, sample current responses of an ND7/23 cell without C1V1 but with LNPs (Method 1). Bottom, sample current responses of another ND7/23 cell expressing C1V1 with out LNPs. (**B**) Summary of current responses to the NIR laser light (975 nm, 58 W/mm^2^). From left to right, column 1: C1V1-null cells with LNPs (n = 7), column 2: C1V1-expressing cells without LNPs (middle, n = 9) and column 3: C1V1-expressing cells with LNPs (right, n = 7). Column 4 shows the summary of current responses to filtered Hg lamp (530–550 nm, 14 mW/mm^2^, n = 7) for reference. The LNPs were applied using Method 1. *P < 0.05 and **P < 0.01 (Kruskal-Wallis test among column 1–3 and Wilcoxon signed-ranks test between column 3 and 4).

Without LNP, only a small magnitude of the inward current was generated by the NIR laser light in a manner dependent on the power (Fig. 3A). The current response was time-dependent, slowly increased during irradiation and slowly decreased upon shutting off the light. A similar inward current was evoked in the control ND7/23 without expressing C1V1 but in the presence of LNP particles. However, their magnitudes were almost negligible, at least within 1 s (Fig. 3B). It is suggested that the green luminescent light emitted from LNPs effectively activated C1V1 to generate a photocurrent.

**Figure 4:**
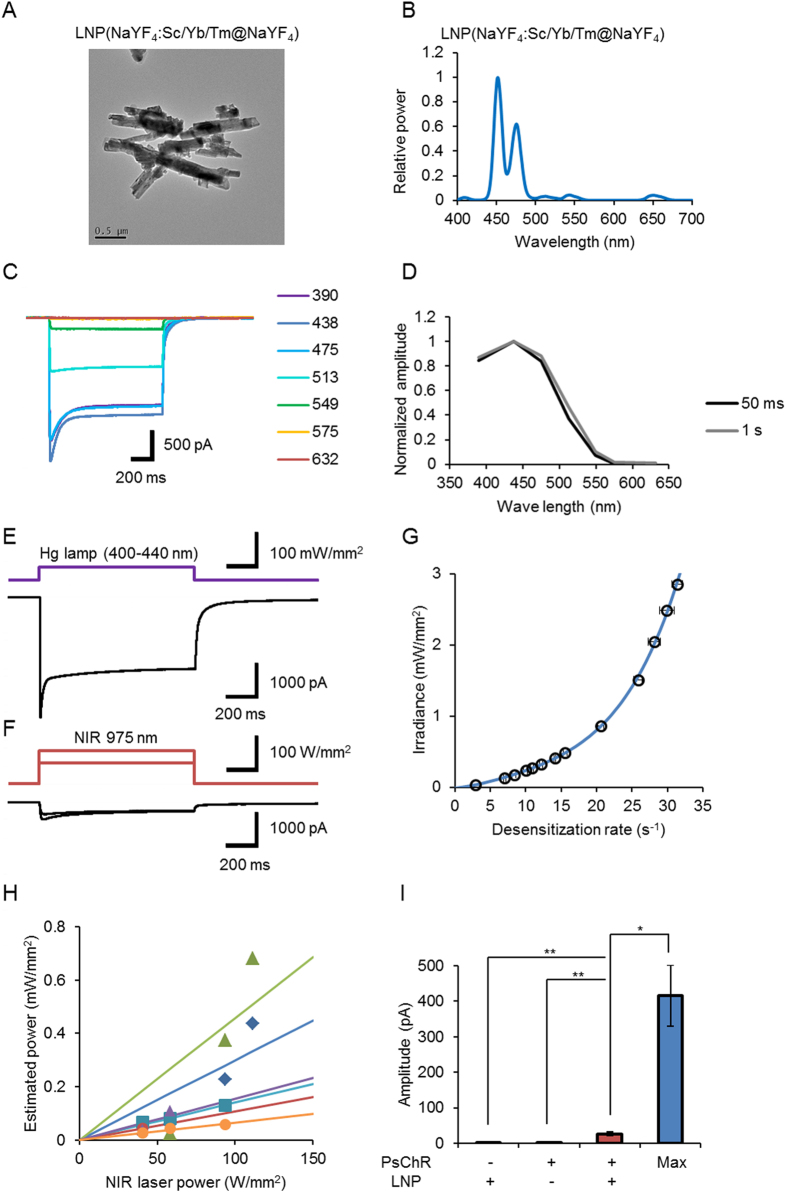
NIR up-conversion activation of PsChR. (**A**) TEM images of LNP(NaYF_4_:Sc/Yb/Tm@NaYF_4_). Scale, 500 nm. (**B**) The relative emission spectrum. (**C**) Sample photocurrents of PsChR evoked by 1-s irradiation of light at 390, 438, 475, 513, 549, 575 and 632 nm (ND7/23 cells). Each power of light was adjusted to 0.5 mW/mm^2^ on the specimen. (**D**) Spectral sensitivity of PsChR photocurrent; charge during initial 50 ms (black) and 1 s (gray). (**E**) A sample photocurrent of PsChR evoked by filtered Hg lamp (400–440 nm, 37 mW/mm^2^) at a holding potential of −60 mV (ND7/23 cells). (**F**) In the same cell, photocurrents were also evoked by the NIR laser light (975 nm, 58 and 94 W/mm^2^, respectively). (**G**) The relationship between desensitization rate of PsChR photocurrent and the irradiance at 438 nm. Note that the ordinate is the irradiance and the abscissa is the desensitization rate. Each symbol is mean ± SEM (n = 6). The blue line is drawn by the least squares fitting of the experimental data to a polynomial function: *y* = 0.0000040*x*^4^ − 0.00011*x*^3^ + 0.0021*x*^2^ + 0.011*x* − 0.013. (**H**) The relationship between the estimated power of blue light emission and the NIR laser power (Method 1). Each symbol represents individual ND7/23 cell. The slope of linear approximation gives the energy efficiency. (**I**) Summary of current responses to the NIR laser light (975 nm, 58 W/mm^2^). From left to right, column 1: PsChR-null cells with LNPs (n = 6), column 2: PsChR-expressing cells without LNPs (middle, n = 5) and column 3: PsChR-expressing cells with LNPs (right, n = 7). Column 4 shows the summary of the current responses to filtered Hg lamp (400–440 nm, 37 mW/mm^2^, n = 7) for reference. The LNPs were applied using Method 1. *P < 0.05 and **P < 0.01 (Kruskal-Wallis test among column 1–3 and Wilcoxon signed-ranks test between column 3 and 4).

In another series of experiments, we used LNP(NaYF_4_:Sc/Yb/Tm@NaYF_4_), which emits its maximum at 450 and 480 nm (Fig. 4A,B), as a donor and one of the ChRs from the marine alga, *Platymonas subcordiformis* (PsChR)^23^, which preferentially absorbs 390–475 nm light, as an acceptor (Fig. 4C). The total charge between 0 and 50 ms peaked at 438 nm and showed similar spectral sensitivity as that between 0 and 1 s (Fig. 4D) due to the relatively small off time constants of PsChR (7.9 ± 0.33 ms, n = 7). When a PsChR-expressing ND7/23 cell was irradiated with the filtered violet light of a Hg lamp (400–440 nm; 37 mW/mm^2^), an almost maximal inward photocurrent was generated with a peak and a plateau at a holding potential of −60 mV (Fig. 4E). The inward currents were also evoked by the NIR laser light (975 nm) in a manner dependent on the power (Fig. 4F). In summary, in the experiments using Method 1, the NIR laser light at 58 W/mm^2^ evoked 6.8 ± 1.5% (n = 7) at the peak and 8.1 ± 2.2% (n = 7) in the steady state of the maximal photocurrent evoked by the filtered Hg lamp. The power of blue light emission was estimated by the desensitization rate in the case of LNP(NaYF_4_:Sc/Yb/Tm@NaYF_4_) because the rising phase of the PsChR photocurrent hardly fitted a simple exponential relationship. As shown in Fig. 4G, the irradiance at 438 nm was experimentally approximated to a fourth order polynomial relationship of the desensitization rate. The estimated power of blue emission was variable from one cell to another but was dependent on the NIR laser power in the same cell (Fig. 4H). Therefore, the energy efficiency using Method 1 was on average 0.20 × 10^−3^ ± 0.060 × 10^−3^% (range, 0.023 × 10^−3^–0.32 × 10^−3^%, n = 7) with relatively large variability (coefficient of variation, 0.72). However, without LNP or PsChR the NIR laser light evoked negligible inward current within 1 s (Fig. 4I). It is suggested that the blue luminescent light emitted from LNPs effectively activated PsChR to generate a photocurrent.

**Figure 5:**
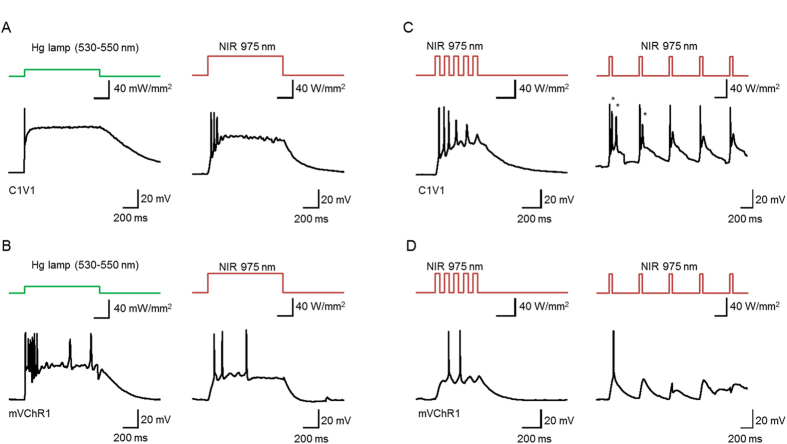
Neuronal activation by up-conversion. (**A**) Sample responses of a C1V1-expressing neuron; left, to the filtered Hg lamp (530–550 nm, 14 mW/mm^2^) and right, to the NIR laser (41 W/mm^2^) with LNP(NaYF_4_:Sc/Yb/Er) using Method 2. (**B**) Similar to A, but from an mVChR1-expressing neuron. (**C**) Sample responses of the same neuron shown in A to 50 ms NIR pulses (975 nm, 41 W/mm^2^) at 10 Hz (left) and 2 Hz (right). (**D**) Sample responses of the same neuron shown in B to 50 ms NIR pulses (975 nm, 41 W/mm^2^) at 10 Hz (left) and 2 Hz (right). *surplus action potentials.

To test the possibility of using the LNP luminescence to control neural activity, we used cultured cortical neurons that expressed C1V1 or mVChR1. The NIR laser light was applied as a train of short pulses of 50 ms to the LNP(NaYF_4_:Sc/Yb/Er) particles using Method 2. Of the seven C1V1-expressing neurons that generated action potentials by the filtered Hg lamp, in 100% action potentials were generated by the NIR laser pulse at 41 W/mm^2^ (Fig. 5A). Similarly, 100% of the mVChR1-expressing neurons generated action potentials by the NIR laser at 41 W/mm^2^ (n = 4) (Fig. 5B). However, the number of action potentials and their frequency were variable from case to case even when the laser pulses were applied at 2–10 Hz (Fig. 5C,D). As shown in Fig. 5C, multiple action potentials were often generated in response to a short pulse of 50 ms in a neuron expressing C1V1 or mVChR1.

## Discussion

In the present study we provide evidence that the NIR light could be applied for the optogenetic manipulation of neural activities when LNPs are used as donors and ChRs as acceptors. Upon NIR irradiation, the LNP(NaYF_4_:Sc/Yb/Er) emitted visible light that in turn activated C1V1 or mVChR1 to generate a photocurrent in the cells. Although the amplitude of the photocurrent was dependent on the power of the NIR laser, only negligible response was evoked by the same laser without LNP or ChR. These small responses are possibly generated by heat as ND7/23 cells naturally have temperature-sensitive TRP channels^24^. The heat effects would thus be minimized by minimizing the laser power and/or the irradiation time. The NIR irradiation of LNPs also depolarized the membrane potential of the C1V1/mVChR1-expressing neurons to evoke action potentials. The variability of the firing pattern could be attributed to the diversity of neuronal membrane characteristics and the magnitude of the photocurrent, neither of which could be controlled in the present study. The generation of surplus action potentials with the laser pulses as short as 50 ms can be attributed to the relatively slow off kinetics of C1V1 or mVChR1. These properties could make the LNP(NaYF_4_:Sc/Yb/Er)-C1V1/mVChR1 system suitable for depolarizing a targeted neuron for a relatively long period with minimal NIR irradiation. The firing pattern of neurons using the LNP(NaYF4:Sc/Yb/Tm@NaYF_4_)-PsChR system should be investigated in the future, since the PsChR photocurrent (off time constant, 7.9 ms) is much faster in its kinetics than C1V1 (150 ms) or mVChR1 (100 ms).

Since the rate of rise of ChR is attributed to the intrinsic molecular dynamics, it is quantitatively related to the irradiance regardless of the cellular expression^3^. Indeed, it almost followed a first order relationship of irradiance when it was relatively small in magnitude^25^,^26^. This enabled us to estimate the power of the green light emitted from the LNPs at a cell. The energy efficiency, which is the quotient of the visible light power received by a cell divided by the NIR laser power, was variable from one cell to another, probably because of the uneven density of LNPs and the diverse geometrical relationship between the recorded cell and the LNPs. That is, the energy efficiency of LNP(NaYF_4_:Sc/Yb/Er) was on average 0.12 × 10^−3^% with Method 1 and 0.029 × 10^−3^% with Method 2 and its variability was larger in Method 1 than in Method 2 (Supplementary Fig. S1 online). Although not significant, the difference between Method 1 and 2 can be attributed to the distance between the LNPs and the recorded cell; indeed, two coverslips (0.12–0.17 mm thickness) were inserted between the LNPs and the cell in the case of Method 2. This distance appeared to make the power of emitted light more even in Method 2 than in Method 1. Similarly, the energy efficiency of the blue light generated by LNP(NaYF4:Sc/Yb/Tm@NaYF_4_) was on average 0.20 × 10^−3^% (Method 1) of the laser power. It is suggested that the different reactions between the LNP(NaYF_4_:Sc/Yb/Er)-C1V1 system and the LNP(NaYF4:Sc/Yb/Tm@NaYF_4_)-PsChR system can be attributed to differences in the population light sensitivities of the acceptor ChRs (Supplementary Fig. S2 online).

When the LNPs were injected in the brain, their luminescence was visible even by eye with the irradiation of the modest NIR light and their chemical toxicity was negligible as reported previously^19^,^31^ (Supplementary Fig. S3 online). The up-conversion system thus has a potential to actuate neurons deep in the brain non-invasively. However, future experiments should clarify optimized conditions for *in vivo* applications of the system. First, the LNPs should be optimized in the spectra as well as the efficiency of luminescence. This could be accomplished by the fabrication of core-shell nanoparticles with layers of different rare earth contents^27^,^28^. Second, the ChRs should be optimized in the action spectra, the kinetics and the sensitivity to light. For example, those with slow off kinetics such as bistable ChR variants^21^,^26^,^29^,^30^ could become sensitive acceptors if the spectral sensitivity can be adjusted to the LNP luminescence. Third, the LNP should be positioned as close as possible to the ChR molecule as the power density is proportional to the reciprocal square of the distance. It is anticipated that the energy transfer would become maximal if the LNP can be tagged to ChR at the molecular level. Fourth, the NIR optics should be optimized to irradiate the targeted neurons effectively with minimal tissue damage by heat. Although small in magnitude, a significant current was generated by NIR light without ChR or LNPs in a manner dependent on time. Therefore, pulsed irradiation would be less harmful to the cell or tissue than the continuous irradiation. Finally, the biosafety of LNPs should be extensively investigated for longer period. The safety could be improved by coating the LNPs with water-soluble and biocompatible ligands^31^,^32^.

Optogenetics may be applied for the treatment of neural dysfunctions such as Parkinson's disease. Deep brain stimulation is one of the most effective treatments for Parkinson’s disease^33^. Indeed its major symptoms were relieved by optogenetic stimulation of the direct pathway of the dorsal striatum in an animal model^34^. These examples illustrate the potential for the LNP-ChR system to increase our understanding of the mechanisms of brain disease and enable us to develop novel therapies for them with at least two advantages. First, the targeted neurons would be able to be irradiated remotely with minimal damage to the brain. Second, the effects of irradiation would be localized to regions close to the LNPs. A variety of biomedical applications of NIR light would thus be open through optimization of the LNP-ChR system.

## Methods

### Synthesis of LNP

To a stirred solution of oleic acid (OA; 20 m*L*) and NaOH (1.2 *g*) in ethanol/H_2_O (20/6 m*L*) was added a mixture of rare earth chlorides (RECl_3_) with a total amount of 0.8 m*mol* in H_2_O (8 m*L*) and a solution of NH_4_F (298.3 m*g*) in H_2_O (4 m*L*). After stirring for 10 min, the mixture was transferred in an autoclave and stirred for 2 h at 200 °C. The mixture was cooled down to room temperature and centrifuged (14,000 rpm) for 15 min to obtained LNPs (NaYF_4_:Sc/Yb/Er). To synthesize NaYF_4_:Sc/Yb/Tm@NaYF_4_, a mixture of YCl_3_ (0.8 m*mol*) in H_2_O (8 m*L*), a solution of NH_4_F (298.3 m*g*) in H_2_O (4 m*L*), and the above-obtained nanoparticle solution (5 m*L*) was added to a stirred solution of OA (20 m*L*) and NaOH (1.2 *g*) in ethanol/H_2_O (20/6 m*L*). After stirring for 10 min, the mixture was transferred to an autoclave and stirred for 2 h at 200 °C. The mixture was cooled down to room temperature and centrifuged (14,000 rpm) for 15 min. The obtained nanoparticles were washed three times with ethanol/H_2_O (1/1) and dried *in vacuo* before use in the biological experiments.

### Luminescence spectra

LNP in H_2_O (1 m*g*/m*L*) was subjected to luminescence measurement using a fluorospectrometer (FP-8500, JASCO, Hachioji, Japan) equipped with an external laser diode excitation source (300 mW, 980 nm, THORLABS, Inc., Newton, NJ).

### TEM measurement

The size and morphology of the LNPs were determined with a JEM-2010 F (JEOL, Tokyo, Japan) field emission transmission electron microscopy (FE-TEM) at 200 kV. A sample solution ultrasonicated for 30 s in isopropanol was droped on a supported grid.

### Expression plasmids

A chimeric channelrhodopsin cDNA encoding *Chlamydomonas reinhardtii* channelrhodopsin-1, ChR1 (GenBank acc. no. AF385748; 1–164th amino acids), and *Volvox carteri* channelrhodopsin-1, VChR1 (GenBank acc. no. EU622855; 121–300th amino acids), was prepared by overlap extension PCR as described previously^35^. In the case of mVChR1, cDNA was designed so that the N-terminal coding sequence (1–23rd amino acids) of VChR1 was replaced with that of ChR1 (1–66th amino acids). The mVChR1 cDNA was commercially synthesized (GenScript, Tokyo, Japan) and subcloned into 6P1-CAG plasmid as previously described^36^. A human codon-optimized PsChR (1–326th amino acids, GenBank acc. no. JX983143) was commercially synthesized (GenScript) and subcloned in-frame into the pVenus-N1.

### Cell culture

The electrophysiological assays of ChR variants were made using ND7/23 cells, hybrid cell lines derived from neonatal rat dorsal root ganglia neurons fused with mouse neuroblastoma^37^. ND7/23 cells were grown on a collagen-coated coverslip in Dulbecco's modified Eagle’s medium (Wako, Osaka, Japan) supplemented with 2.5 μM all-*trans* retinal, 10% fetal bovine serum under a 5% CO_2_ atmosphere at 37 °C. The expression plasmids were transiently transfected in ND7/23 cells using Effectene Transfection Reagent (Qiagen, Tokyo, Japan) according to the manufacturer’s instructions. Electrophysiological recordings were then conducted 16–48 h after the transfection. Successfully transfected cells were identified by the presence of Venus fluorescence.

To apply LNPs to ND7/23 cells we adopted two methods (Fig. 2B). In the case of Method 1, 10 mg LNPs were mixed with 1 m*L* collagen solution (50 μ*g* collagen/m*L* 0.01 N acetic acid). A round coverslip (diameter, 12 mm) was coated with this mixture (about 30 μ*L*/piece) and then cured by overnight incubation. After washing with water 3 times, the ND7/23 cells were seeded on the coat then cultured. In the case of Method 2, the coverslip was coated with a mixture (about 30 μ*L*/piece) of LNPs and silicone resin (5 m*g*/m*L*, SILPOT 184, Dow Corning Toray, Tokyo, Japan), and then cured. The LNP-coated coverslip was put underneath the specimens upside down in the recording chamber.

Cortical neurons were isolated from embryonic day 16 Wistar rats (Japan SLC Inc., Shizuoka, Japan) using Nerve-Cells Dispersion Solutions (Sumitomo Bakelite, Tokyo, Japan) according to the manufacturer's instructions and grown in culture medium (Sumitomo Bakelite) under a 5% CO_2_ atmosphere at 37 °C. The expression plasmids were transiently transfected in cortical neurons using calcium phosphate transfection method at days *in vitro* (DIV)5 or 6. Electrophysiological recordings were then conducted at DIV21–23 in neurons identified to express Venus fluorescence under a conventional epifluorescence system. To apply LNPs to primary cultured neurons we adopted Method 2 as the coated collagen was often peeled off from the coverslip during the relatively long culturing period in the case of Method 1.

### Electrophysiology

All experiments were carried out at room temperature (23 ± 2 °C). Photocurrents were recorded as previously described^3^ using an EPC-8 amplifier (HEKA Electronic, Lambrecht, Germany) under a whole-cell patch clamp configuration. The data were filtered at 1 kHz and sampled at 10 kHz (Digdata1440 A/D, Molecular Devices Co., Sunnyvale, CA) and stored in a computer (pClamp10.3, Molecular Devices).

The internal pipette solution for the whole-cell voltage-clamp recordings from ND7/23 cells contained (in mM) 120 KOH, 100 glutamate, 5 EGTA, 50 HEPES, 2.5 MgCl_2_, 2.5 MgATP, 0.1 Leupeptin, 0.01 Alexa568, adjusted to pH 7.3 with KOH. The internal pipette solution for the whole-cell current-clamp recordings from cortical neurons contained (in mM) 125 K-gluconate, 10 KCl, 0.2 EGTA, 10 HEPES, 1 MgCl_2_, 3 MgATP, 0.3 Na_2_GTP, 10 Na_2_-phosphocreatine, 0.1 Leupeptin, adjusted to pH 7.2 with KOH. The extracellular artificial cerebrosipinal fluid (ACSF) contained (in mM) 125 NaCl, 2.5 KCl, 25 NaHCO_3_, 1.25 NaH_2_PO_4_, 2.5 CaCl_2_, 1.25 MgCl_2_, 11 glucose, equilibrated with mixed gas containing 95% O_2_ and 5% CO_2_.

### Optics

Irradiation was carried out using a filtered Hg lamp (peak, 530–550 nm or 400–440 nm) and near-infrared (NIR) fiber-coupled CW laser (975 nm, FL-FCSE, Focuslight Technologies, Xi’an, China) controlled by computer software (pCLAMP10.3, Molecular Devices). The optic fiber (diameter, 50 μm) was guided in the patch electrode as close as 5.0 mm from its tip. To investigate the action spectrum, irradiation was carried out using SpectraX light engine (Lumencor Inc., Beaverton, OR) controlled by computer software (pCLAMP10.3, Molecular Devices) at wavelengths (nm, >90% of the maximum): 390 ± 18, 438 ± 24, 475 ± 28, 513 ± 17, 549 ± 15, 575 ± 25 and 632 ± 22.

The power of the NIR laser was directly measured by a laser power meter (407A, Spectra-Physics, Tokyo, Japan) through the optical fiber and patch electrode, and divided by the luminescent area of the LNP sheet positioned at the tip of the patch electrode (Supplementary Fig. S4 online). The power of visible light was directly measured under microscopy by a visible light-sensing thermopile (MIR-100Q, Mitsubishi Oil Chemicals, Tokyo, Japan) and was adjusted to 0.5 mW/mm^2^ on the specimen. Every photocurrent was measured with a holding potential of −60 mV and at pH 7.4 outside.

### Numerical and statistical analysis

Data analysis was performed with Clampfit 10.3 (Molecular Devices). To measure the rate of rise of the photocurrent, a single exponential function was fitted to the activation phase for digital points at 10 kHz between 10 and 90% of the maximum. To measure the desensitization rate, a single exponential function was fitted to the desensitization phase for digital points at 10 kHz between 10 and 90% of the maximum. Each linear or non-linear regression analysis was performed using a least-squares fitting algorithm (*R*, R Development Core Team, 2005). All data in the text and figures are expressed as mean ± SEM and were evaluated with the Kruskal-Wallis test for statistical significance, unless otherwise noted. It was judged as statistically insignificant when P > 0.05.

## Additional Information

**How to cite this article**: Hososhima, S. *et al.* Near-infrared (NIR) up-conversion optogenetics. *Sci. Rep.* **5**, 16533; doi: 10.1038/srep16533 (2015).

## Supplementary Material

Supplementary Information
