# Supplementary material for: Near-infrared (NIR) up-conversion optogenetics
Source: Sci Rep. 2015 Nov 10;5:16533. doi: 10.1038/srep16533 (PMC4639720; doi:10.1038/srep16533)
Supplement: Supplementary Information [file srep16533-s1.docx]

**Supplementary Information to**

**Near-infrared (NIR) up-conversion optogenetics**

Shoko Hososhima, Hideya Yuasa, Toru Ishizuka, Mohammad Razuanul Hoque, Takayuki Yamashita, Akihiro Yamanaka, Eriko Sugano, Hiroshi Tomita

and Hiromu Yawo


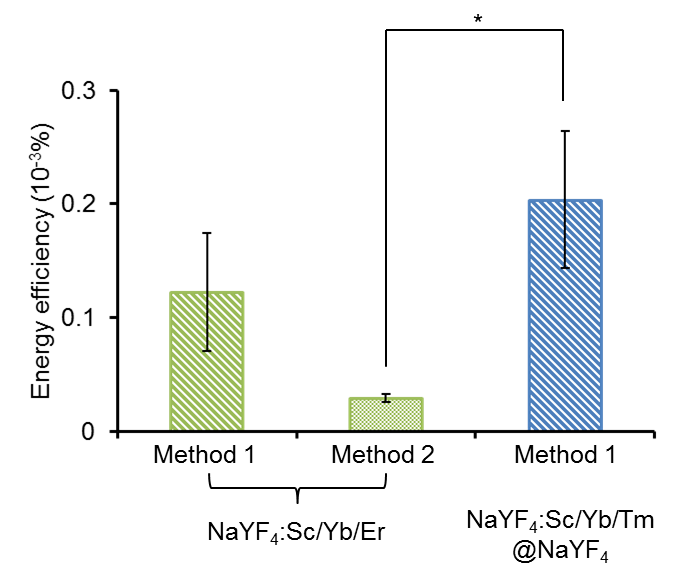


**Supplementary Figure S1 | Summary of the energy efficiency.** The energy efficiency is the quotient of the visible light power at a cell divided by the NIR laser power. The LNP(NaYF_4_:Sc/Yb/Er)-C1V1 system with Method 1 (left), the LNP(NaYF_4_:Sc/Yb/Er)-C1V1 system with Method 2 (middle) and the LNP(NaYF4:Sc/Yb/Tm@NaYF_4_)-PsChR system with Method 1 (right). *, P < 0.05 (Kruskal-Wallis test).


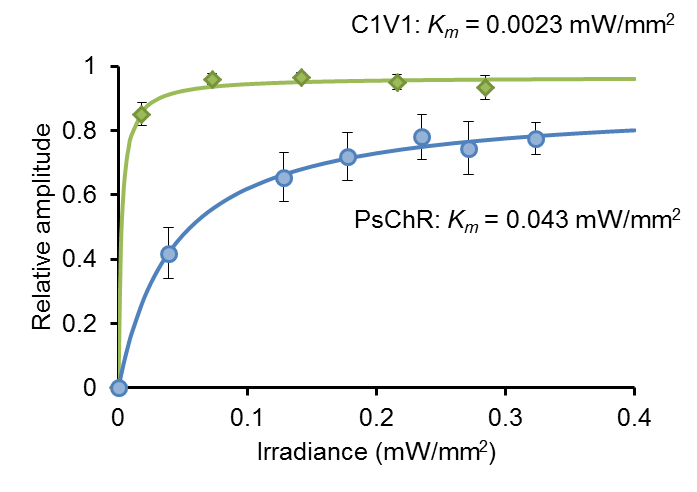


**Supplementary Figure S2 | The population light sensitivities of acceptor ChRs.** The relative peak amplitude of photocurrent as a function of irradiance: C1V1 (green diamond, n = 5) and PsChR (blue circle, n = 6). The photocurrents were measured from the expressed ND 7/23 cells at a holding potential of -60 mV in response to green light (549 nm) and blue light (438 nm), respectively. Each relationship was fitted to a simple Michaelis-Menten kinetics: *y = I*_max_ (*x /* ( *x + K*_m_ ) ), where *x* is the irradiance, *y* is the relative amplitude. Parameters, *I*_max_ and *K*_m_ were determined by a least-squares fitting algorithm (*R*, R Development Core Team, 2005): 0.97 and 0.0023 mW/mm^2^ for C1V1, 0.89 and 0.043 mW/mm^2^ for PsChR.


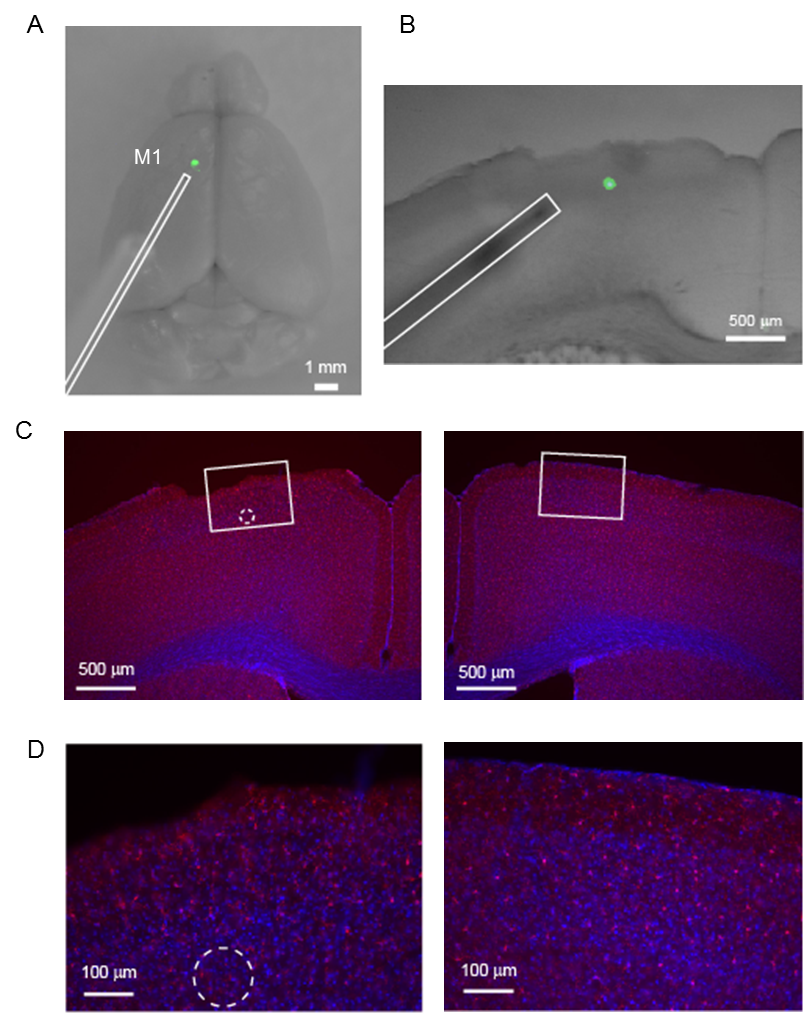


**Supplementary Figure S3 | The relationship between the NIR laser current and its power density at the specimen.** The power of NIR laser was directly measured by a laser power meter through an optical fiber and patch electrode, and divided by the luminescent area of the LNP sheet positioned at the tip of the patch electrode (red triangles). The distance between the fiber tip and the LNP sheet was 5 mm. The red line is the least-squares approximation with the relationship, *y* = 35 *x* – 12.


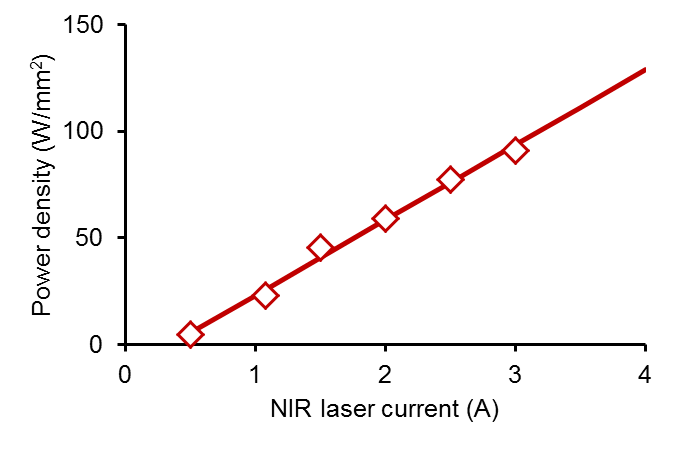


**Supplementary Figure S4 | The relationship between the NIR laser current and its power density at the specimen.** The power of NIR laser was directly measured by a laser power meter through an optical fiber and patch electrode, and divided by the luminescent area of the LNP sheet positioned at the tip of the patch electrode (red triangles). The distance between the fiber tip and the LNP sheet was 5 mm. The red line is the least-squares approximation with the relationship, *y* = 35 *x* – 12.
